# Inpatient Treatment Outcome in a Large Sample of Adolescents with Anorexia Nervosa

**DOI:** 10.3390/nu15194247

**Published:** 2023-10-02

**Authors:** Norbert Quadflieg, Silke Naab, Sandra Schlegl, Tabea Bauman, Ulrich Voderholzer

**Affiliations:** 1 Department of Psychiatry and Psychotherapy, LMU University Hospital, Ludwig-Maximilians-University Munich (LMU), 80336 Munich, Germany; sandra.schlegl@med.uni-muenchen.de (S.S.); uvoderholzer@schoen-klinik.de (U.V.); 2 Schoen Clinic Roseneck Affiliated with the Medical Faculty of the University of Munich (LMU), 83209 Prien, Germany; snaab@schoen-klinik.de (S.N.); tbauman@schoen-klinik.de (T.B.); 3 Department of Psychiatry and Psychotherapy, University Hospital, 79106 Freiburg, Germany

**Keywords:** adolescents, anorexia nervosa, eating disorder, treatment, CBT-E, outcome

## Abstract

Anorexia nervosa is an illness affecting primarily adolescent girls and young women. Clinical guidelines recommend early intervention, with inpatient treatment for more severe cases. We present an evaluation of a multi-modal cognitive–behavioral inpatient treatment (CBT-E) involving carers in specialized units for adolescents. Routine data of 962 adolescent inpatients (26 boys) (mean age 15.48 [1.26]; range 12–17 years) were analyzed. Predictors of good body weight outcome (achieving a discharge BMI of at least 18.5 kg/m^2^) were identified by logistic regression analysis. Mean inpatient treatment lasted 96.69 (45.96) days. The BMI increased significantly from 14.93 (1.38) kg/m^2^ at admission to 17.53 (1.58) kg/m^2^ at discharge (z = 26.41; *p* < 0.001; d = 1.708). Drive for thinness decreased from 29.08 (9.87) to 22.63 (9.77; z = 18.41; *p* < 0.001; d = 0.787). All other subscores of the Eating Disorder Inventory also decreased significantly, with small to medium effect sizes. General psychopathology also showed significant decreases. The Beck Depression Inventory-II score decreased from 26.06 (11.74) to 16.35 (12.51; z = 18.41; *p* < 0.001; d = 0.883). A good body weight outcome was predicted by a higher BMI at admission (OR = 1.828), age at onset at 15 years or higher (OR = 1.722), and higher Somatization (OR = 1.436), Anxiety (OR = 1.320), and Bulimia (OR = 1.029) scores. CBT-E involving carers is an efficient intervention for adolescents with anorexia nervosa.

## 1. Introduction

Anorexia nervosa (AN) is a life-threatening eating disorder (ED). The main clinical symptom is a self-induced loss of body weight resulting in a low body mass index (BMI) of less than 18.5 kg/m^2^ associated with an intense fear of gaining weight and a preoccupation with body weight and shape. This condition not only negatively affects health-related quality of life [1], but also affects later young adulthood [2], causing concerns in near relatives and family members [3]. Diagnostic manuals describe two basic types of AN. Patients with a restrictive type of AN try to achieve low weight exclusively through restrictive eating, fasting, or an increase in expenditure of energy (burning of calories) by excessive exercise and movement. Patients with a binge-eating/purging type of AN have eating binges and experience a loss of control overeating, even when consuming only small amounts of food. This type of AN also includes purging behaviors like self-induced vomiting or the use of laxatives, diuretics, and other substances to prevent weight gain or to achieve weight loss [4].

The most frequent onset of AN is in childhood and adolescence [5]. This is a period involving a high risk for developing an ED. Physical changes during puberty have effects on self-esteem and body dissatisfaction, and they are a potential cause for developing AN. However, other significant psychosocial and biological changes occur in the same time period, and the role of pubertal changes is not clear [6].

To avoid a poor course and chronification of AN, current clinical guidelines recommend early intervention [7]. Treatment options include outpatient, inpatient, or day-clinic treatment, as well as—more recently—home treatment [8]. The choice of the treatment setting is usually determined by symptom severity, comorbidity, and the national health system and its associated costs. All treatment options have to address the restoration of normal weight, medical problems, the psychological symptoms of AN (fear of gaining weight, fixation on body weight and shape, and food), as well as comorbid psychiatric and personality disorders [9]. Inpatient treatment for adolescents with AN is recommended for health conditions including very low body weight, low energy intake by solid and fluid food, severe comorbidity and medical conditions, and severe psychosocial impairment [7].

A number of previous studies supported the effectiveness of inpatient treatment for AN in adolescents. However, sample size was usually low, varying between 11 and 74 participants [10,11,12,13,14,15,16,17,18]. More recently, two studies reported larger sample sizes of 126 [19] and 289 [20] adolescents with AN. An additional study included 218 adolescents with AN in a mixed adolescent and adult sample, but it did not report results for adolescents separately [21]. The results of these studies will be described in more detail in the discussion of the present study.

Treatment outcome in a sample of 238 female adolescent inpatients of the Schoen Clinic Roseneck with AN was reported by Schlegl et al. [22]. This study found a significant increase in body weight and improvement of ED-specific and non-ED specific psychopathology during treatment. Multivariate linear regression analysis identified longer inpatient treatment, lower age, and no previous inpatient treatment as correlates or predictors of and increase in BMI. The present study reports the treatment outcome in adolescent inpatients with AN with the aim to expand the sample of Schlegl et al. [22] to a very large number of adolescent inpatients, with a sample size larger than the combined sample size from other inpatient studies, in order to provide reliable data on the effectiveness of cognitive–behavioral inpatient psychotherapy (CBT-E). A second aim was to identify predictors of good body weight outcome, applying a BMI threshold indicative of weight recovery to the normal range.

## 2. Materials and Methods

### 2.1. Participants

Between August 2013 and December 2020, 1123 adolescents aged between 12 and 17 years were admitted to treatment for AN at the Schoen Clinic Roseneck in Prien, Germany. For 161 patients, the BMI at discharge could not be extracted from the database. Thus, 962 adolescents (26 boys [2.7%]; age 15.48 [1.26]) provided information on body weight at admission and discharge and were included in this study. ICD-10 diagnoses included F50.00 (restrictive AN; N = 864, 89.8%), F50.01 (AN binge-eating/purging type; N = 52, 5.4%), and F50.1 (atypical AN; N = 46, 4.8%). The number of patients was 66 in age group 12/13 years, 393 in age group 14/15 years, and 503 in age group 16/17 years at the time of treatment. Mean inpatient treatment for the included patients lasted 96.69 (45.96) days.

### 2.2. Treatment

Patients were treated in specialized ED units for adolescents by a multidisciplinary team of psychologists, physicians, co-therapists, and professionals from other disciplines (e.g., nutritionists). All treatment was voluntary in open units. Patients in need of compulsory treatment were not admitted to the clinic. Psychotherapy included a manualized ED coping training consisting of multimodal disorder-specific and age-adapted CBT-E with individual and group sessions. The nine group therapy sessions included behavior and functional analysis, ED-symptom-maintaining cognitions, dealing with emotions and needs, relapse prevention, acceptance of one’s body, and psychoeducation. All ED behaviors were considered in therapy, including binge-eating and purging behaviors. The frequency of therapy sessions was one to three times a week for group sessions (90 min), and individual therapy (50 min) once or twice a week. In addition, treatment included social skills training, art therapy, therapeutically supervised cooking training, and gradually expanded elements of sports and movement therapy depending on the physical condition and weight gain of the patients. At the beginning of therapy, three therapeutically supervised meals per day were obligatory, and patients with AN were required to gain 500–1000 g per week following the recommendations of the German S3-guidelines [7]. Patients were weighed twice a week on average by the clinical staff and weight gain was visualized on charts. If patients did not gain weight, food intake requirements were increased, and food intake was monitored during mealtimes. In case of problems with excessive exercise, the activity levels were gradually reduced. In rare, extreme cases, high caloric fluids and nasal tube feeding were administered. Patients were discharged from inpatient treatment after having improved in body weight, medical factors, and comorbid conditions. Discharge could also be initiated by the patient, often against the advice of the therapists. Discharges by the clinic for disciplinary breaches were also possible. Another reason for discharge could be that the patient’s health insurance would no longer cover the costs of inpatient treatment.

Parents or other carers were involved through telephone consultations and at least three family therapy sessions [23]. They also provided information on the development of actual ED symptoms and other behaviors of the patient, which was included in the clinical documentation and used in the psychotherapy of the patient.

### 2.3. Assessment Instruments

As part of the routine intake procedure, patients answered a questionnaire package, which included the following assessments.

(1) The Eating Disorder Inventory-2 (EDI-2) [24,25] assessed ED symptoms and psychological features in 11 subscales with higher scores indicating more symptom severity (Cronbach’s α at admission in our sample indicated in parentheses): Drive for Thinness (α = 0.92), Bulimia (α = 0.87), Body Dissatisfaction (α = 0.87), Ineffectiveness (α = 0.89), Perfectionism (α = 0.77), Interpersonal Distrust (α = 0.81), Interoceptive Awareness (α = 0.86), Maturity Fears (α = 0.76), Asceticism (α = 0.79), Impulse Regulation (α = 0.81), and Social Insecurity (α = 0.79).
(2) The Brief Symptom Inventory (BSI) [26,27] assessed general psychopathology in nine subscales: Somatization (α = 0.82), Obsessive–Compulsive Symptoms (α = 0.81), Interpersonal Sensitivity (α = 0.81), Depression (α = 0.86), Anxiety (α = 0.79), Anger–Hostility (α = 0.69), Phobic Anxiety (α = 0.76), Paranoid Ideation (α = 0.72), and Psychoticism (α = 0.75), with higher values indicating more psychopathology.
(3) The Beck Depression Inventory-II (BDI-II) [28] provides a single sum score of Depression (α = 0.92) with higher scores indicating more depression.
(4) Information on sociodemographic characteristics, pretreatment, height, weight, and psychiatric comorbidity were taken from the hospital documentation.

### 2.4. Statistical Analyses

Means and standard deviations at admission and discharge, or frequencies, are reported. In testing for statistically significant differences, non-parametric Wilcoxon and Mann–Whitney U-tests for repeated measures and comparison of independent groups (respectively) with a critical *p*-value of *p* < 0.05 were applied. All analyses included both boys and girls. Effect sizes are reported as Cohen’s d with small effects indicated by d = 0.2–0.5, medium effects by d = 0.5–0.8, and large effects by d > 0.8 [29]. For comparing the change of the BMI over treatment between different age groups, repeated measures analysis of variance on the ranks with post-hoc pairwise comparison was used. Partial eta-square (η^2^) of 0.01, 0.06, and 0.14 indicate small, medium, and large effect sizes in this analysis, respectively [29]. Not all patients filled out the questionnaires at discharge, reducing the number of cases for comparison of admission and discharge values. The exact number of cases included is reported for each analysis.

Reaching a BMI of at least 18.5 kg/m^2^ at discharge defined a good body weight outcome. Predictors of a good body weight outcome were identified by logistic regression analysis (0 = poor, 1 = good body weight outcome) using stepwise forward selection. Potential predictors were available from the routine assessment at admission and included age, sex, age at onset, pretreatment, BMI, the subscales of the EDI-2, BDI-II score, the subscales of the BSI, and the presence of psychiatric comorbidity (yes/no).

## 3. Results

The 161 patients, mentioned above as not included in the study did not differ from the 962 participants in age (15.42 [1.26] versus 15.48 [1.26] years, z = 0.575, *p* = 0.565, d = 0.051) and BMI (15.08 [1.44] kg/m^2^ versus 14.93 [1.38] kg/m^2^, z = 1.230, *p* = 0.219, d = 0.105) at admission, but had a shorter duration of treatment (75.02 [49.83] versus 96.69 [45.96] days, z = 4.883, *p* < 0.001, d = 0.466).

### 3.1. Eating Disorder Symptom Outcome

**Figure 1:**
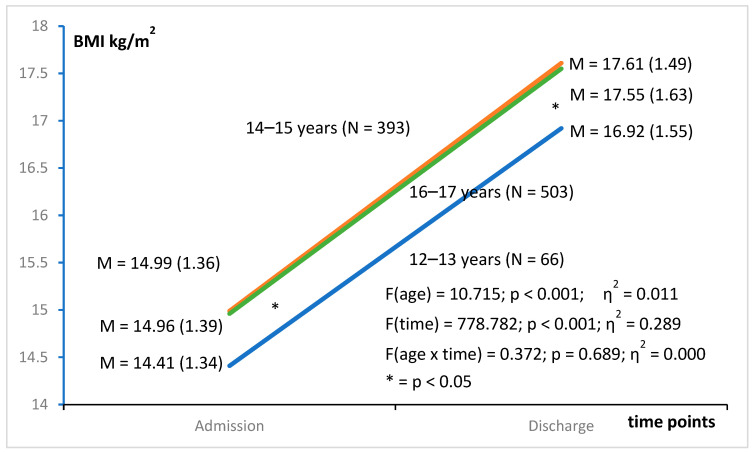
Increase in body mass index during inpatient treatment in three age groups of adolescents with anorexia nervosa.

The BMI increased significantly from 14.93 (1.38) kg/m^2^ to 17.53 (1.58) kg/m^2^ over treatment (z = 26.409, *p* < 0.001) with a high effect size (d = 1.708). Average weight gain per week was 570 (682) g. The increase in BMI was similar in age groups 12–13 years, 14–15 years, and 16–17 years. The two older groups showed a nearly identical course, differing significantly from the youngest age group (*p* < 0.05). The interaction of time and age group, however, was not statistically significant (Figure 1).

**Figure 2:**
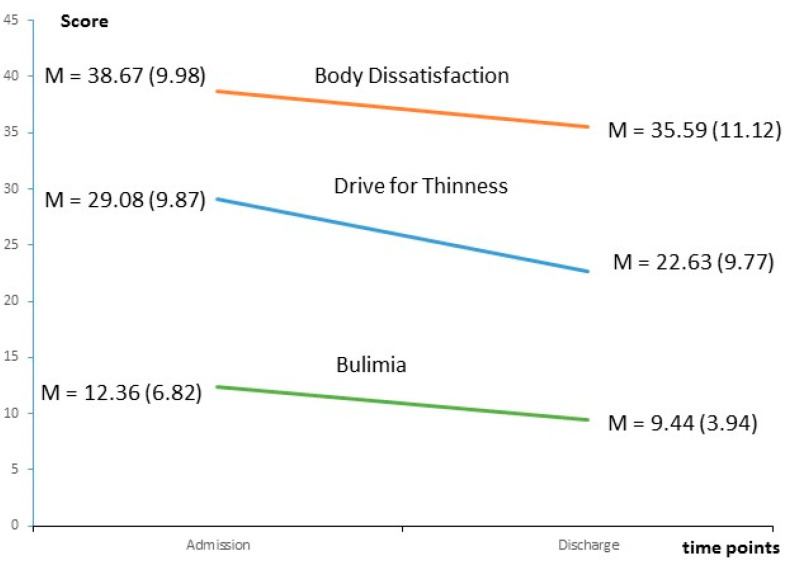
Change of ED-specific subscale scores of the Eating Disorder Inventory-2 from admission to discharge in adolescent inpatients with anorexia nervosa. All changes from admission to discharge were significant (*p* < 0.05).

Table 1 gives an overview of the EDI-2 subscale scores at admission and discharge. All scores decreased significantly (*p* < 0.001) over treatment with small to medium effect sizes. For the most important ED-specific subscales, this is also illustrated in Figure 2. Drive for Thinness showed a steeper decrease in symptom severity than Bulimia and Body Dissatisfaction.

### 3.2. General Psychopathology Outcome

Table 2 lists the scores of the BSI and BDI-II at admission and discharge. All scores decreased significantly (*p* < 0.001) over treatment, with the BSI scores showing small to medium effect sizes. A high effect size (d = 0.883) was observed for the BDI-II score.

### 3.3. Comparison of Eating Disorder and General Psychopathology in Patients with Good and Poor Body Weight Outcomes

Patients with good body weight outcomes were not necessarily free from other symptoms of mental disorder. Table 3 lists the BMI and the EDI-2 scores separately for patients with good and poor body weight outcomes. With the exception of Interpersonal Distrust and Maturity Fears (*p* > 0.05), patients with good body weight outcomes reported more severe ED symptoms (EDI-2) at the beginning of treatment than patients with poor body weight outcomes (*p* < 0.05). At the end of treatment, patients in both outcome groups had converged to more similar and lower EDI-2 scores. Drive for Thinness, Body Dissatisfaction, Interoceptive Awareness, and Impulse Regulation still differed at the end of treatment between outcome groups, with more symptom severity in the good body weight outcome group. All effect sizes were small.

Table 4 compares the BSI and the BDI-II scores of patients with good and poor body weight outcomes. Essentially, the pattern described for EDI-2 scores was repeated for the BSI and BDI-II. Scores in the good body weight outcome group at the beginning of the treatment showed more severity in patients with good body weight outcomes compared to patients with poor body weight outcomes. Most of the severity scores still differed between outcome groups at the end of treatment, with higher scores in the good body weight outcome group. Again, all effect sizes were small.

### 3.4. Predictors of Good Body Weight Outcome

Results of the final logistic regression model are presented in Table 5. A higher BMI, later age at onset (15 years or higher), and higher BSI Somatization, BSI Anxiety, and EDI-2 Bulimia scores at admission to inpatient treatment increased the probability of reaching a BMI of at least 18.5 kg/m^2^ at discharge from inpatient treatment. A total of 263 patients (27.3%) reached this good body weight outcome.

## 4. Discussion

According to our knowledge, the present study includes, by far, the largest adolescent sample with inpatient treatment for AN. It reports treatment outcome and adds substantially to the evaluation of multimodal inpatient treatment, primarily based on CBT-E, in adolescents with AN, which also involved carers. Highest effect sizes were found for core ED symptoms with lower effect sizes for general psychopathology. ED symptoms were reduced significantly during treatment, with high effect sizes for weight increase and reduction in Drive for Thinness. Although improving during treatment, effect sizes for personality traits Perfectionism and Interpersonal Distrust were low, highlighting important topics to be addressed in subsequent outpatient psychotherapy. However, our questionnaires covered only the severity of these personality traits. We did not assess if and to what extent our therapy improved the ability to cope with consequences of these personality traits in everyday life outside the clinic. Sharing their life experience informally with other patients may also have contributed to better coping. This informal sharing of life experience between patients is a specific characteristic of inpatient treatment that merits further investigation.

Depression, anxiety, and other psychopathology also decreased significantly during treatment. Restoration to normal weight was predicted by a higher BMI at the beginning of treatment, a later age at AN onset of at least 15 years, and higher Somatization, Anxiety, and Bulimia scores at the beginning of treatment.

The duration of inpatient treatment in our adolescent sample was, on average, 96.69 days, or 13.81 weeks. Other German studies of adolescents with AN reported a somewhat longer mean inpatient stay of 14.6 weeks in a randomized controlled trial [14] and a longer median inpatient stay of 17 weeks in a multi-center adolescent AN registry study [20]. A study from Italy reported a similar length of stay of 13 weeks, with an additional 7 weeks of day hospital treatment [11]. Another study from France reported a longer inpatient treatment of 19 weeks [30], with a similar length of stay (20 weeks) reported in a sample from Scotland [16]. In a sample treated in England, length of stay averaged 15 weeks [31]. Considerably shorter inpatient stays were reported for adolescents with AN in Spain (30 days) [10], the United States of America, and Canada (11 to 50 days [32]; 11 days [33]; 26 days in a mixed adolescent/adult sample [34]; 38 days [35]). Similarly, median lengths of stay of 17 days for the year 2019 and 12 days for the year 2020 were reported by an Australian study [36]. Summarizing, the duration of inpatient treatment of our study was comparable to other European studies with the exception of Spain. Much shorter inpatient stays were reported for the North American continent and for Australia. These differences are mainly attributable to differences in the national health care systems. Striegel-Moore et al. [34] argue that short inpatient treatments are not sufficient for achieving weight restoration. This supports the usefulness of longer inpatient stays for the treatment of AN. On the other hand, the mean BMI at discharge from inpatient treatment in our study was about 17.50 kg/m^2^. This means that a part of the patients remained below this often-used threshold of 17.50 kg/m^2^ for the diagnosis of AN and did not achieve weight restoration. However, the primary aim of inpatient treatment of AN is to give an impetus for improvement to very severe AN cases, and not weight restoration. Having improved in body weight and other medical factors, the indication for inpatient treatment is no longer present, and the patient has to be returned or newly referred to outpatient treatment. As a rule, health insurances will not bear the costs of inpatient treatment beyond what is absolutely necessary. A more recent proposal is home treatment following inpatient treatment. This recommendable treatment was shown to be suitable for maintaining or achieving target weight [37].

Concerning body weight, several studies reported results similar to our study. In these studies, the BMI at the beginning of inpatient treatment varied between 14.40 kg/m^2^ and 15.50 kg/m^2^ [10,14,19,20] which is near the admission BMI in our study (14.93 kg/m^2^). In some studies, the BMI increased significantly during inpatient treatment, from 15.5 kg/m^2^ to 18.4 kg/m^2^ and 18.6 kg/m^2^ [10,20], respectively, which is also similar to our findings, although the level of the values is higher than in our sample. This may indicate a more severe ED in our sample. In another study, an increase in BMI from 14.40 kg/m^2^ to 17.01 kg/m^2^ was reported [19]. These lower values may be attributable to the more severe BMI inclusion threshold of 17.50 kg/m^2^ in this study. This study also reported an effect size of d = 2.01, higher than in our sample. Possibly, the lower BMI at the beginning of treatment allowed a steeper trajectory of increase in body weight.

Mairhofer et al. [19] reported changes of EDI-2 subscales during inpatient treatment. Although it is not stated explicitly in the publication, it seems that Mairhofer et al. applied a recoding procedure to the answer codes as proposed in the original publication of the Eating Disorder Inventory. This modification reduces the original 1–6 answer format to a 0–3 format, explaining the lower scores. Our study followed the instructions of the EDI-2 manual, building the scores from the format 1–6 without recoding [25]. Consequently, we will confine the comparison with our results to statistical significance and effect sizes. Both the present study and the study of Mairhofer et al. [19] reported significant improvement (*p* < 0.05) for EDI-2 subscales Drive for Thinness, Bulimia, Interpersonal Distrust, Interoceptive Awareness, Maturity Fears, Asceticism, Impulse Regulation and Social Insecurity. Improvement of EDI-2 subscales Body Dissatisfaction, Ineffectiveness, and Perfectionism was significant in the present study but not in the study of Mairhofer et al. [19]. Both studies point in the same direction and support the efficacy of multi-modal inpatient treatment for improving cognitive symptoms of AN in adolescents. The discrepancy regarding statistical significance may well reflect the increased statistical power of our larger sample of 962 vs. 126 inpatients in the sample of Mairhofer et al.

Efficacy of the inpatient CBT-E in our sample was also confirmed by the results of general psychopathology scores. The significant improvement for all assessments is supported by the findings of Dalle Grave et al. [11], which reported a significant decrease in the BSI-general symptomatic or severity index. This study also supports our finding of the efficacy and acceptance of inpatient CBT-E in adolescents treated for AN.

Dalle Grave et al. [11] also provided follow-up data for their sample of 26 inpatients after CBT-E treatment. In this study, weight gain and reduction in severity of ED and general psychopathology was maintained for over 12 months after treatment. Similarly, a 12-month follow-up in a small subgroup of our present sample showed a further increase in body weight and maintenance of the improved ED severity and depression one year after inpatient treatment [38]. Both our study and the study of Dalle Grave et al. [11] allow for the conclusion that CBT-E is a suitable inpatient treatment of adolescent AN, improving body weight, negative thoughts, body image, and other important areas. An additional study reported a BMI above 17.5 kg/m^2^ in 80.4% of adolescents 7.5 years after inpatient treatment but a low quality of mental health [39].

Concerning the predictors of good body weight outcome, we did not confirm the findings of Schlegl et al. [22], although both studies shared part of the sample. One possible reason is the different approach to the outcome criterion (continuous variable of BMI change versus a dichotomous BMI threshold). The earlier study considered any BMI change, while the present study defined a good body weight outcome threshold. Another possible reason is that Schlegl et al. [22] included the length of inpatient treatment in the predictive model. In a strict sense, this is not a predictor, as it is not precedent to treatment outcome. Including this variable limits the comparability of the results. In the present study, a higher BMI at the beginning of treatment increased the probability of good body weight outcome. This is in agreement with other studies, which reported a higher BMI at admission predicting good post-treatment weight maintenance [10], and a lower BMI predicting drop-out from inpatient treatment in a mixed sample of adolescent and adult patients [21].

Age at onset of AN at 15 years or higher also increased the probability of a good body weight outcome. This is still an early age at onset and, considering the age limit of our study of less than 18 years, this finding may reflect a shorter duration of AN, and thus a timelier intervention. Regrettably, in the hospital documentation, age at onset was only stored in categories, preventing the computation of the duration of AN in this sample. Another possibility is that these slightly older patients were more mature in their cognitive and mental development, and, thus, were able to gain more benefit from the therapy. The finding of lower age as a predictor of increase in BMI over treatment by Schlegl et al. [22] is contrary to our result. However, the approach to the prediction of outcome was different in these studies. Schlegl et al.’s outcome criterion was the change of BMI values in a multivariate linear regression analysis. This approach included negative and positive as well as small and large changes of BMI in a continuous outcome variable. The present study used a fixed BMI threshold as the outcome criterion and summarized negative and positive increases in BMI in the same category of poor outcome, as long as the BMI threshold of 18.5 kg/m^2^ was not reached, ignoring the absolute value and sign of BMI change. Considering the known influence of sample and predictor selection on the results of regression analyses, the inclusion of the length of inpatient stay by Schlegl et al. may have also impacted the correlation between predictors. In the study of Herpertz-Dahlmann et al. [39], patients below age 12 did not have a worse outcome than older adolescent patients. This also contradicts the findings of Schlegl et al. [22] that lower age is correlated to increase in body weight.

Higher Anxiety and Somatization scores at admission also increased the probability of very good body weight outcome. In addition to these significant predictors, all EDI-2 and BSI scores were higher in patients with good body weight outcomes compared to patients with poor body weight outcomes in our adolescent sample, but the effect sizes were small. Fichter et al. [40] compared EDI-2 and BSI scores between the good and poor outcome groups in a predominantly adult sample and found only EDI-2 Perfectionism and EDI-2 Maturity Fears scores to differ between groups. Some scores at the beginning of treatment were also higher in the long-term good outcome group [40]. A possible explanation of these findings is an enhanced motivation to change and increased responsiveness to interventional measures in more anxious adolescents. This points to the usefulness of early intervention before a learning process of successful maintenance of negative cognitions about one’s body sets in, which could lead to chronification and an enduring course of AN.

The final predictor of good body weight outcome was a higher Bulimia score. As one of the more tangible behavioral symptoms of AN, this symptom may well offer a good starting point for intervention suitable for establishing at least a preliminary working alliance of patient and therapist. Results from a study on predominantly adult female inpatients with AN, however, show contrary results. In a 2-year follow-up, neither binging nor purging behaviors were predictors of outcome, and, in a 6-year follow-up, binging behavior was a predictor of poor outcome, but purging behavior was no predictor [41]. In a similar study, the EDI-2 Bulimia score was no predictor of outcome in a 10-year follow-up [40].

Several limitations have to be considered in interpreting the results: (1) As in most studies on AN, nearly all patients included in the present study were females, and our results may not apply to male adolescent inpatients. However, our intention was to present a “natural” sample as it occurs in the practice of a specialized clinic. The percentage of boys in our sample (2.7%) was very similar to the percentage of adolescent boys in the sample collected in a patient registry by Jaite et al. (2.3%) [20]; (2) Patients were from one clinic only and it is not known if the results apply to patients in other clinics; (3) Excluding the BMI, assessments were made by self-report questionnaires. However, as the focus of our research was on change of symptoms, the same assessments were used at the beginning and at the end of treatment; (4) Very little is known on predictors of outcome in adolescents with AN. Consequently, our interpretation of predictors is partially speculative and needs further support from other studies; (5) Our outcome definition focused on body weight restoration. While this is a central symptom with possible severe impact on the physical condition of the patient, this definition ignores other important symptoms of AN like the cognitive focus on body weight and shape, slimness ideal, and fear of gaining weight, as well as emotional conditions like depressive symptoms. Additional areas not covered by our outcome definition are the social and occupational functioning of the patients. From the data in the hospital documentation, we could not derive measures like, for example, the Morgan Russell Outcome Assessment Schedule [42], which is suitable for describing outcome in much more detail; (6) Patients improved while being in the clinic, but we have no data on how persistently this improvement could be maintained after returning to everyday life. However, results from a follow-up in a small subgroup of our sample showed a longer-lasting effect of CBT-E after the end of treatment [38]; (7) We could not extract detailed data on comorbidity, excessive exercise, and medication, limiting our set of potential predictors of outcome. This is an important future research question.

These limitations are offset by a number of strengths of our study: (1) The sample size is extraordinarily large, resulting in reliable estimates of symptom change; (2) Only variables assessed before the outcome were included in the search for real predictors of outcome; (3) Good body weight outcome was defined to reflect a normal body weight at the end of treatment.

## 5. Conclusions

Concluding, inpatient multi-modal CBT-E is effective in the treatment of severe AN. While weight restoration is important in AN, focusing on ED symptoms is not sufficient, and still more specific psychotherapy for personality traits like Perfectionism and Interpersonal Distrust, as well as comorbid symptoms of depression, anxiety, etc., is needed. Further research should address which elements of multi-modal CBT-E are the most important contributors to treatment success. The contributions of the family and carers to the improvement of AN also needs additional research.

## Figures and Tables

**Table 1:** Change of body mass index and Eating Disorder Inventory-2 scores over treatment in adolescents with anorexia nervosa.

	Admission	Discharge		Statistics	
	Mean (SD)	Mean (SD)	*z*-Value	*p*-Value	d
Body Mass Index kg/m^2^ N = 962	14.93 (1.38)	17.53 (1.58)	26.409	<0.001	1.708
Drive for Thinness N = 807	29.08 (9.87)	22.63 (9.77)	18.406	<0.001	0.787
Bulimia N = 806	12.36 (6.82)	9.44 (3.94)	14.430	<0.001	0.519
Body Dissatisfaction N = 794	38.67 (9.98)	35.59 (11.12)	8.816	<0.001	0.342
Ineffectiveness N = 763	35.33 (10.46)	31.48 (10.88)	11.522	<0.001	0.446
Perfectionism N = 803	20.24 (6.27)	19.35 (6.12)	5.465	<0.001	0.191
Interpersonal Distrust N = 802	22.71 (6.44)	21.76 (6.58)	5.125	<0.001	0.176
Interoceptive Awareness N = 800	33.74 (10.00)	28.80 (10.33)	14.110	<0.001	0.560
Maturity Fears N = 784	28.71 (7.16)	26.44 (7.34)	10.718	<0.001	0.406
Asceticism N = 775	24.38 (7.63)	21.14 (7.56)	12.982	<0.001	0.523
Impulse Regulation N = 805	26.44 (8.22)	24.16 (8.33)	9.735	<0.001	0.348
Social Insecurity N = 803	27.63 (6.89)	25.61 (7.39)	9.758	<0.001	0.351

**Table 2:** Change of Brief Symptom Inventory and Beck Depression Inventory-II (BDI-II) scores over treatment in adolescents with anorexia nervosa.

	Admission	Discharge		Statistics	
	Mean (SD)	Mean (SD)	*z*-Value	*p*-Value	d
Somatization N = 828	0.99 (0.80)	0.56 (0.58)	16.002	<0.001	0.599
Obsessive–Compulsive Symptoms N = 828	1.36 (0.91)	0.92 (0.79)	14.080	<0.001	0.533
Interpersonal Sensitivity N = 827	1.59 (1.05)	1.26 (1.00)	9.439	<0.001	0.353
Depression N = 828	1.50 (1.00)	1.04 (0.95)	13.472	<0.001	0.501
Anxiety N = 828	1.09 (0.81)	0.82 (0.72)	10.623	<0.001	0.369
Anger–Hostility N = 828	0.98 (0.71)	0.62 (0.63)	13.845	<0.001	0.509
Phobic Anxiety N = 827	0.62 (0.74)	0.44 (0.69)	7.861	<0.001	0.250
Paranoid Ideation N = 828	0.97 (0.78)	0.84 (0.78)	5.100	<0.001	0.167
Psychoticism N = 828	1.24 (0.90)	0.85 (0.85)	12.504	<0.001	0.465
BDI-II Score N = 690	26.06 (11.74)	16.35 (12.51)	18.209	<0.001	0.883

**Table 3:** Body mass index and Eating Disorder Inventory-2 scores in patients with poor and good body weight outcomes.

		Admission			Discharge	
	Poor Outcome Mean (SD)	Good Outcome Mean (SD)	Mann–Whitney z-Value d	Poor Outcome Mean (SD)	Good Outcome Mean (SD)	Mann–Whitney z-Value d
Body Mass Index kg/m^2^ N = 699/263/699/263	14.66 (1.32)	15.65 (1.26)	10.156 *p* < 0.001 d = 0.756	16.89 (1.30)	19.25 (0.72)	23.931 *p* < 0.001 d = 2.018
Drive for Thinness N = 650/239/626/246	28.36 (10.08)	31.80 (9.03)	4.890 *p* < 0.001 d = 0.350	22.13 (9.92)	23.60 (9.66)	2.096 *p* = 0.036 d = 0.149
Bulimia N = 649/240/625/246	11.71 (6.29)	14.41 (7.96)	4.813 *p* < 0.001 d = 0.397	9.20 (3.76)	9.88 (4.12)	1.895 *p* = 0.058 d = 0.177
Body Dissatisfaction N = 644/239/616/246	38.12 (10.10)	41.04 (9.62)	3.705 *p* < 0.001 d = 0.293	35.27 (10.96)	35.70 (11.98)	0.766 *p* = 0.044 d = 0.038
Ineffectiveness N = 624/233/606/242	34.77 (10.49)	38.08 (10.18)	4.120 *p* < 0.001 d = 0.318	31.09 (10.69)	32.28 (11.01)	1.452 *p* = 0.146 d = 0.110
Perfectionism N = 651/239/623/245	20.16 (6.34)	21.19 (5.98)	2.163 *p* = 0.031 d = 0.164	19.10 (6.19)	19.92 (5.94)	1.820 *p* = 0.069 d = 0.134
Interpersonal Distrust N = 647/240/623/246	22.65 (6.55)	23.57 (6.24)	1.921 *p* = 0.055 d = 0.141	21.66 (6.57)	22.07 (6.65)	0.707 *p* = 0.479 d = 0.062
Interoceptive Awareness N = 650/236/622/246	33.03 (10.33)	37.09 (8.98)	5.378 *p* < 0.001 d = 0.406	28.25 (10.43)	30.37 (10.18)	2.979 *p* = 0.003 d = 0.205
Maturity Fears N = 637/237/619/245	28.45 (7.29)	29.37 (6.70)	1.894 *p* = 0.058 d = 0.130	26.40 (7.35)	26.87 (7.08)	1.044 *p* = 0.297 d = 0.064
Asceticism N = 629/241/611/240	24.06 (7.85)	26.19 (7.36)	3.732 *p* < 0.001 d = 0.276	20.90 (7.56)	21.68 (7.34)	1.622 *p* = 0.105 d = 0.104
Impulse Regulation N = 650/242/622/245	25.87 (8.08)	28.75 (8.20)	4.500 *p* < 0.001 d = 0.355	23.69 (8.16)	25.42 (8.71)	2.664 *p* = 0.008 d = 0.208
Social Insecurity N = 646/242/620/245	27.45 (7.09)	28.83 (6.49)	2.542 *p* = 0.011 d = 0.199	25.43 (7.33)	26.24 (7.49)	1.262 *p* = 0.207 d = 0.111

**Table 4:** Brief Symptom Inventory and Beck Depression-Inventory-II (BDI-II) scores in patients with poor and good body weight outcomes.

		Admission			Discharge	
	Poor Outcome Mean (SD)	Good Outcome Mean (SD)	Mann–Whitney z-Value d	Poor Outcome Mean (SD)	Good Outcome Mean (SD)	Mann–Whitney z-Value d
Somatization N = 662/246/632/241	0.92 (0.77)	1.20 (0.84)	4.635 *p* < 0.001 d = 0.347	0.54 (0.59)	0.65 (0.61)	2.872 *p* = 0.004 d = 0.188
Obsessive–Compulsive Symptoms N = 662/246/632/241	1.29 (0.89)	1.60 (0.92)	4.634 *p* < 0.001 d = 0.347	0.90 (0.79)	1.02 (0.83)	2.133 *p* = 0.033 d = 0.158
Interpersonal Sensitivity N = 662/246/631/241	1.55 (1.06)	1.87 (1.02)	4.304 *p* < 0.001 d = 0.312	1.23 (0.99)	1.39 (1.03)	2.217 *p* = 0.027 d = 0.164
Depression N = 662/246/632/241	1.46 (1.00)	1.78 (0.99)	4.309 *p* < 0.001 d = 0.321	1.01 (0.93)	1.18 (0.99)	2.376 *p* = 0.018 d = 0.181
Anxiety N = 662/246/632/241	1.04 (0.77)	1.31 (0.89)	4.050 *p* < 0.001 d = 0.336	0.80 (0.74)	0.89 (0.71)	2.071 *p* = 0.038 d = 0.113
Anger–Hostility N = 662/246/632/241	0.92 (0.67)	1.18 (0.80)	4.216 *p* < 0.001 d = 0.367	0.61 (0.62)	0.69 (0.71)	1.076 *p* = 0.282 d = 0.126
Phobic Anxiety N = 661/246/632/241	0.58 (0.73)	0.81 (0.80)	4.826 *p* < 0.001 d = 0.302	0.42 (0.69)	0.56 (0.74)	2.814 *p* = 0.005 d = 0.197
Paranoid Ideation N = 662/246/632/241	0.95 (0.78)	1.09 (0.79)	2.620 *p* = 0.009 d = 0.176	0.84 (0.79)	0.90 (0.77)	1.490 *p* = 0.136 d = 0.076
Psychoticism N = 662/246/632/241	1.22 (0.93)	1.42 (0.88)	3.376 *p* < 0.001 d = 0.221	0.84 (0.85)	0.91 (0.86)	1.178 *p* = 0.239 d = 0.082
BDI-II N = 606/226/560/223	25.13 (11.86)	29.58 (11.09)	4.741 *p* < 0.001 d = 0.382	15.36 (12.25)	17.90 (13.15)	2.525 *p* = 0.012 d = 0.203

**Table 5:** Predictors of good body weight outcome in adolescents treated for anorexia nervosa.

No.	Predictor	Odds Ratio	95% Confidence Interval	Wald- Statistics	*p*
1	Higher body mass index	1.828	1.566–2.134	58.346	<0.001
2	Age at onset at 15 years or higher	1.722	1.167–2.540	7.512	0.006
3	Higher BSI Somatization score	1.436	1.092–1.887	6.724	0.010
4	Higher BSI Anxiety score	1.320	1.016–1.714	4.319	0.038
5	Higher EDI-2 Bulimia score	1.029	1.003–1.056	4.830	0.028

R^2^ = 0.23, N = 686. Note: All predictors were assessed at admission to inpatient treatment (df = 1). EDI-2 = Eating Disorder Inventory-2, BSI = Brief Symptom Inventory. Good body weight outcome was defined as the patient achieving a BMI at discharge from inpatient treatment of 18.5 or higher. An odds ratio above 1 means an increase in the probability of good body weight outcome.

## Data Availability

In accordance with the data security guidelines of our facility, the Schön Klinik Roseneck, data relating to patients must not be publicly accessible.
